# Optimizing Ultrasound Probe Disinfection for Healthcare-Associated Infection Control: A Comparative Analysis of Disinfectant Efficacy

**DOI:** 10.3390/microorganisms12122394

**Published:** 2024-11-22

**Authors:** Gaetano Ferrara, Giovanni Cangelosi, Sara Morales Palomares, Stefano Mancin, Marianna Melina, Orejeta Diamanti, Marco Sguanci, Antonella Amendola, Fabio Petrelli

**Affiliations:** 1 Nephrology and Dialysis Unit, Ramazzini Hospital, 41012 Carpi, Italy; amaranto1984@libero.it; 2 Units of Diabetology, ASUR Marche, 63900 Fermo, Italy; giovanni.cangelosi@virgilio.it; 3 Department of Pharmacy, Health and Nutritional Sciences (DFSSN), University of Calabria, 87036 Rende, Italy; sara.morales@unical.it; 4 IRCCS Humanitas Research Hospital, Via Manzoni 56, Rozzano, 20089 Milan, Italy; 5 Azienda Socio Sanitaria Territoriale Lariana, 22100 Como, Italy; mariannamelina@tiscali.it; 6 Veneto Institute of Oncology IOV-IRCCS, 35128 Padua, Italy; orejeta.diamanti@iov.veneto.it; 7 A.O. Polyclinic San Martino Hospital, Largo R. Benzi 10, 16132 Genova, Italy; sguancim@gmail.com; 8 Department of Health Sciences, Università Degli Studi di Milano, 20146 Milan, Italy; antonella.amendola@unimi.it; 9 School of Pharmacy, Polo Medicina Sperimentale e Sanità Pubblica “Stefania Scuri”, Via Madonna delle Carceri 9, 62032 Camerino, Italy; fabio.petrelli@unicam.it

**Keywords:** ultrasound probes, disinfection, healthcare-associated infections, pathogen transmission, infection control, review

## Abstract

**Background/Aims:** Ultrasound is a key diagnostic tool in modern medicine due to its ability to provide real-time, high-resolution images of the internal structures of the human body. Despite its undeniable advantages, there are challenges related to the contamination of ultrasound probes, with the risk of healthcare-associated infections. The aim of this review was to identify the most effective disinfectants for disinfecting ultrasound probes to prevent the transmission of pathogens between patients. **Methods:** A narrative review was conducted using the PubMed, CINAHL, Embase, and Cochrane Library databases, resulting in the inclusion of 16 studies from an initial 1202 records. **Results:** Hydrogen peroxide (H_2_O_2_) was the most effective disinfectant, especially in automated systems, achieving a >5-log_10_ reduction in viral load, including that of resistant pathogens like *Human Papillomavirus*. Chlorhexidine gluconate (4%) demonstrated strong antibacterial efficacy, eliminating 84.62% of bacterial contamination, but was less effective against viral pathogens. Glutaraldehyde was effective in some cases, though its use carried a higher risk of probe damage. The use of sodium hypochlorite varied across guidelines; some endorsed it for COVID-19 prevention, while others cautioned against its application due to potential probe damage. **Conclusions:** This study highlights the importance of advanced disinfection technologies and strict adherence to protocols in improving infection control. Automated systems utilizing H_2_O_2_ strike an ideal balance between antimicrobial efficacy and equipment preservation. Future research should focus on developing disinfection methods that prioritize safety, cost-effectiveness, and environmental sustainability in various clinical environments.

## 1. Introduction

Ultrasound is a key diagnostic tool in modern medicine, valued for its ability to provide real-time, high-resolution images of the body’s internal structures [1]. Due to its versatility, non-invasiveness, and safety, ultrasound has become indispensable across numerous clinical disciplines, including radiology, gynecology, cardiology, and emergency medicine [2]. The principle of ultrasound involves the emission of high-frequency sound waves that reflect off body tissues based on density. These reflected waves are then converted into detailed images, allowing the visualization of internal organs, blood vessels, muscles, and other tissues. Unlike X-rays or computed tomography (CT), ultrasound uses no ionizing radiation [3]. Technological advances have also led to more portable and powerful devices. Ultrasound is used not only for diagnosing pathological conditions but also to guide minimally invasive procedures, such as biopsies and drainages, improving precision and reducing risks [4].

In the field of gynecology and obstetrics, ultrasound plays a crucial role in monitoring fetal development and assessing maternal health. It allows for the detection of structural anomalies, the monitoring of placental positioning, and the evaluation of amniotic fluid volume, all of which are critical to fetal well-being [5,6,7,8]. Technological advancements, including the introduction of three-dimensional (3D) and four-dimensional (4D) ultrasound, have further improved its ability to visualize complex anatomical details without exposing the fetus to ionizing radiation, making this method preferable to imaging techniques like X-rays and CT [9].

In cardiology, ultrasound, or echocardiography, is essential for evaluating the heart’s structure and function, including the cardiac valves and myocardial walls. Using Doppler technology, it measures blood flow and helps diagnose conditions like valvular stenosis, heart failure, and cardiomyopathies [9,10]. In emergency medicine, ultrasound plays a vital role in providing rapid, accurate diagnostics at the bedside, known as Point-of-Care Ultrasound (PoCUS). This technique is used in cases of abdominal trauma for Focused Assessment with Sonography for Trauma (FAST) exams and to assess pneumothorax, pleural effusions, and cardiac tamponade, enabling immediate diagnosis and improving patient outcomes [11,12]. Ultrasound also has widespread applications in other clinical areas, such as in abdominal and urological exams. It aids in diagnosing liver disease, gallstones, pancreatitis, and abdominal aortic aneurysms [13]. In urology, it is used to examine the kidneys, bladder, and prostate, diagnosing kidney stones, hydronephrosis, and tumors while guiding procedures like prostate biopsies with precision [14]. In the musculoskeletal field, ultrasound helps diagnose tendon injuries, joint inflammation, and bursitis, allowing dynamic examinations during specific movements to improve diagnostic accuracy [15]. Additionally, ultrasound is widely used in vascular diagnostics. Combined with Doppler technology, it assesses blood circulation and diagnoses conditions like deep vein thrombosis, arterial stenosis, and aneurysms [16,17]. Beyond diagnostics, ultrasound is a key tool for guiding invasive procedures, such as central venous catheter placement or pericardiocentesis, enhancing safety and reducing complications. Its portability and ability to provide immediate results make it indispensable, especially in emergency settings [18].

Despite its advantages, ultrasound probes can become contaminated, posing a risk for healthcare-associated infections. These essential diagnostic tools are a significant transmission risk [19]. When ultrasound probes come into direct contact with patients, they can harbor pathogenic microorganisms, promoting cross-infection, particularly in high-use environments [20]. The risk of transmission increases when probes come into contact with mucous membranes or compromised skin, or are used in invasive procedures such as transvaginal, transrectal, or intraoperative ultrasounds. Inadequate disinfection can lead to the transfer of pathogens between patients or into the environment [21]. Cross-contamination is a well-known source of healthcare-associated infections, contributing to prolonged hospital stays, higher healthcare costs, and elevated morbidity [22]. Studies show that improperly cleaned ultrasound probes can harbor bacteria, viruses, and fungi, posing a serious threat to immunocompromised patients [23]. Antibiotic-resistant organisms like methicillin-resistant *Staphylococcus aureus* (MRSA) can colonize probes, surviving for long periods on moist surfaces, thus increasing the risk of infection [24]. Furthermore, biofilms that form on probes create an additional layer of risk, as they are resistant to standard disinfection techniques [25]. To mitigate this risk, international guidelines advocate for the high-level disinfection of probes that come into contact with mucous membranes or non-intact skin, along with the use of disposable covers [26]. Educating healthcare workers on infection control practices is essential in preventing the spread of healthcare-associated infections. Ultrasound probes used in invasive procedures, such as transvaginal and transrectal exams, are especially prone to contamination [27]. Transvaginal probes, which come into contact with female genital mucous membranes, can be contaminated by bacteria, viruses, or fungi, which can be transmitted to subsequent patients if disinfection is insufficient [28]. Similarly, transrectal probes, which come into contact with the intestinal microbiota, are frequently contaminated with *Escherichia coli* and *Enterococcus faecalis*, pathogens that can cause urinary and systemic infections [29]. Probes used on broken skin also pose a high risk, as microorganisms present can easily penetrate through wounds [30].

Indirect contamination of ultrasound probes can occur through contact with non-sterile instruments, contaminated surfaces, or non-sterile ultrasound gel [31]. Contaminated gel can harbor antibiotic-resistant bacteria such as *Pseudomonas aeruginosa*, known for causing severe infections in hospital settings [32]. *Staphylococcus aureus*, frequently isolated from ultrasound probes, can lead to severe infections, especially in immunocompromised patients [33]. Other opportunistic bacteria, such as *Escherichia coli* and *Pseudomonas aeruginosa*, pose significant risks under favorable conditions, particularly due to their resistance to antibiotic treatments [34]. Viruses such as *Herpes simplex* (HSV) and *hepatitis B* and *C viruses* (HBV, HCV) can be transmitted through contact with infected mucous membranes or contaminated bodily fluids, leading to potentially severe consequences for patients [35]. Additionally, fungi like those of the genus Candida can be transmitted via contaminated probes, posing a heightened risk to immunocompromised individuals [35,36].

In the context of infection management and contamination prevention, it is essential to distinguish between two fundamental concepts: cleaning and disinfection. While cleaning removes dirt through mechanical or physical action using detergents or cleaning agents, disinfection goes a step further by reducing the microbial load on surfaces, materials, and equipment [37]. Effective disinfection is especially crucial for items like ultrasound probes, which, if improperly cleaned, can serve as reservoirs for bacteria, viruses, and fungi, posing a serious threat to patient safety, particularly for immunocompromised individuals. Despite its importance, various disinfectants are available, each with different antimicrobial properties, contact times, and effects on probes. Identifying the most effective disinfectant that eliminates pathogens without damaging probes is key to minimizing cross-infection risks.

### Review Objective

The objective of this review is to identify the most effective disinfectants for ultrasound probes to prevent the transmission of pathogens between patients. This study aims to compare various disinfectants by evaluating their antimicrobial efficacy. The expected outcome is to identify one or more disinfectants that maximize patient safety while preserving the functionality of ultrasound probes.

## 2. Methods

### 2.1. Study Design

A narrative literature review was conducted following the methodology outlined in a previously published study [38], to provide a comprehensive, state-of-the-art review. The aim of this review is to summarize research on the topic, highlighting key developments and shifts in understanding over time.

### 2.2. Identification of the Research Question

The research question for this review was formulated using the PICO model [39], which has been widely used in recent studies [40,41]: “What is the most effective disinfectant for ultrasound probes in preventing pathogen transmission?”. The PICO elements are as follows: P (Population): ultrasound probes; I (Intervention): application of a specific disinfectant; C (Comparison): comparison between different disinfectants; O (Outcome): prevention of pathogen transmission.

### 2.3. Inclusion and Exclusion Criteria

The inclusion criteria included primary and secondary studies published in English and Italian, focusing on the use of disinfectants for ultrasound probes. The literature search was conducted with a time restriction, including only studies published in the last ten years. Studies that were not available in full-text or were not relevant to the research question were excluded.

### 2.4. Search Strategy

The literature search was conducted in August 2024, starting with the PubMed database, followed by searches in the Cumulative Index to Nursing and Allied Health Literature (CINAHL), Embase, and Cochrane Library databases. The keywords used included the following: “disinfectant”, “ultrasound probes”, and “pathogen transmission”, which were combined using Boolean operators (AND, OR). Additional searches were conducted of gray literature sources through the Google Scholar database. Following the initial search to identify relevant records, two academic researchers (M.M. and S.M.) conducted the article selection process. In cases of disagreement, a third researcher (F.V.) was involved to reach a consensus. EndNote 20 was used for managing the bibliographic records (©2024 Clarivate) [42]. To ensure up-to-date scientific evidence, the search was limited to studies from the past 10 years. The complete search algorithms are available in the Supplementary File.

### 2.5. Data Extraction and Synthesis

The selected studies underwent a rigorous two-phase analysis. Initially, they were categorized according to several criteria: Author/Year, Country, Study Design, Sample, Ultrasound Type, Objective, Disinfectant, Disinfection Method, and Results. This categorization provided a systematic approach to synthesizing the identified literature. Subsequently, a narrative synthesis was conducted, supplemented by tables and figures for better visualization and understanding.

## 3. Results

### 3.1. Literature Screening

**Figure 1:**
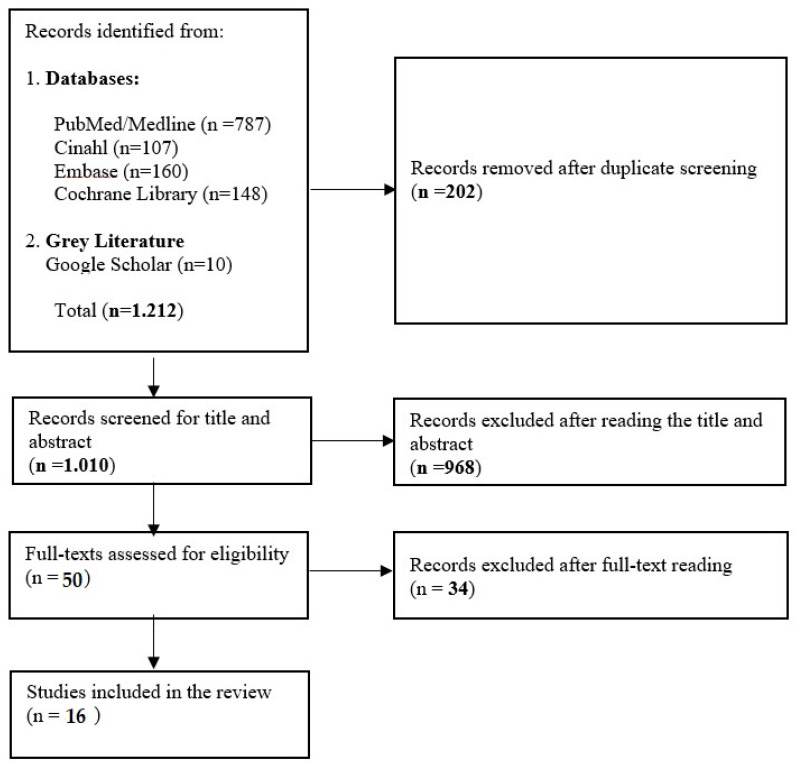
Flow chart of included record selection.

A total of 1202 records were identified through electronic database searches: PubMed/Medline (n = 787), Embase (n = 160), CINAHL (n = 107), Cochrane Library (n = 148), and 10 additional records from gray literature sources via the Google Scholar database. After removing 202 duplicates, 1010 records remained for evaluation. Following a review of titles and abstracts, 968 records were deemed irrelevant, leaving 50 records for full-text screening. At this stage, 34 articles were excluded for not meeting the defined inclusion criteria, resulting in 16 records being included in this narrative review (Figure 1).

### 3.2. General Characteristics of the Studies Included

The sixteen included studies exhibit heterogeneous characteristics [32,43,44,45,46,47,48,49,50,51,52,53,54,55,56,57]. Studies were conducted in various countries, including the United States (n = 3), Australia (n = 2), Thailand (n = 1), the United Kingdom (n = 2), Turkey (n = 1), and France (n = 3). Additionally, three guidelines were included: one from Australia and two from the United States (n = 2). In total, 16 articles were included in this narrative review. Of these, two in vitro experimental studies were included, six were experimental studies, which represented the primary research design, five adopted an observational design, and three were guidelines. The general characteristics of the sixteen studies included in this review are presented in Table 1.

### 3.3. Disinfectant Efficacy and Infection Prevention

Three studies [47,48,49] have emphasized the importance of using effective disinfectants to prevent infections during ultrasound probe use. A common point among these studies is the recommendation to use H_2_O_2_, particularly when combined with thorough preliminary cleaning, to ensure the effectiveness of the disinfection process. The studies by M’Zali et al. [54] and Casalegno et al. [52] analyzed various disinfectant solutions and came to similar conclusions. M’Zali et al. [54] tested the effectiveness of low-level disinfectants (LLDs), such as quaternary ammonium compounds and chlorhexidine, on endovaginal probes. These disinfectants were effective against various bacteria and fungi but showed lower effectiveness against viruses, such as HPV. For this reason, the study suggested that a more potent approach may be necessary, particularly for probes that come into contact with mucous membranes. Similarly, Casalegno et al. [51] examined the use of wipes containing quaternary ammonium compounds to disinfect endocavity probes. While these disinfectants reduced bacterial contamination, high-risk HPV (HR-HPV) contamination was detected in 3% of cases, suggesting that although disinfectants are generally effective, viral contamination may persist.

However, some differences have emerged between guidelines. For example, the study by Basseal JM et al. [48] recommended the use of sodium hypochlorite as a hospital disinfectant, especially for disinfecting surfaces that come into contact with patients infected with COVID-19, while another study [47] advised against using sodium hypochlorite, citing the risk of damaging sensitive ultrasound probes. This discrepancy could be attributed to the different types of infections considered in the two studies: one focuses primarily on disinfection for infections like COVID-19 [48], while the other concentrates on preventing HPV transmission on transvaginal probes, where glutaraldehyde has been recommended as an effective disinfectant. In another study [49], more general guidelines for probe disinfection were provided, including various disinfectants such as quaternary ammonium compounds, H_2_O_2_, and sodium hypochlorite, but excluding glutaraldehyde. The study also classified probes into three categories based on the level of disinfection required: non-critical probes, which come into contact with intact skin, require low-level disinfection (LLD); semi-critical probes, which come into contact with mucous membranes or non-intact skin, require high-level disinfection (HLD); and critical probes, used in sterile body cavities, must be sterilized before use.

Five studies [32,43,44,45,46] have underscored the importance of advanced disinfection methods, such as automated devices that use sonicated H_2_O_2_, like the Trophon^®^ EPR system, and other HLD methods, to effectively reduce contamination, particularly against resistant pathogens like HPV. In these studies, H_2_O_2_ has proven to be at least as effective as, if not more effective than, glutaraldehyde in reducing viral contamination from HPV, highlighting the importance of choosing the right disinfectant based on the type of pathogen. In particular, one study [44] compared the effectiveness of H_2_O_2_ and glutaraldehyde, showing that H_2_O_2_ provides a similar or superior reduction in contamination compared to glutaraldehyde, even against highly resistant viruses like HPV.

The study by Ienghong K et al. [45] compared four cleaning and disinfection methods, testing the effectiveness of dry paper, liquid soap, 4% chlorhexidine gluconate, and dimethyl ammonium chloride. The results showed that all four methods significantly reduced bacterial contamination, with 4% chlorhexidine gluconate being particularly effective, with 84.62% of samples showing no bacterial growth. Additionally, after using dimethyl ammonium chloride or 4% chlorhexidine gluconate, no bacteria, such as coagulase-negative *Staphylococcus* or *Bacillus* spp., were detected, suggesting that these disinfectants may be effective in removing resistant bacteria.

Similarly, another study [43] compared the effectiveness of two HLD methods for ultrasound probes. It was observed that the Trophon^®^ EPR system, which uses sonicated H_2_O_2_, reduced the infectivity of HPV16 and HPV18 by more than 5-log_10_, showing significantly better effectiveness compared to ortho-phthalaldehyde (Cidex^®^ OPA), which achieved an infectivity reduction of less than 0.6-log_10_. These results suggest that automated devices like Trophon^®^ EPR can be essential tools for significantly reducing viral contamination, particularly for resistant pathogens.

A multicenter study conducted in 46 healthcare facilities in France examined their disinfection practices for transvaginal ultrasound probes and ultrasound keyboard surfaces, highlighting adherence to national guidelines for preventing HPV transmission [46]. While 68% of operators correctly followed personal protective measures (wearing gloves and changing them between patients) and 86% correctly protected the ultrasound keyboard from contamination, the results showed that only 2% of cases fully adhered to all recommended stages (probe disinfection, keyboard protection, hand hygiene). A problematic aspect highlighted by the study was that only 8% of operators performed hand hygiene correctly before disinfecting the probe, a crucial step in preventing cross-contamination.

Despite these findings, the use of disposable probe covers and coupling gels was generally in line with best practices. This suggests that while some infection prevention aspects are well-integrated into daily practice, significant improvements are still needed in hand hygiene and in the complete disinfection of surfaces, such as the probe handle and the ultrasound keyboard, which are often not properly sanitized.

Finally, the study by Siyez et al. [53] examined the effectiveness of povidone-iodine in reducing infectious complications in patients undergoing transrectal ultrasound-guided prostate biopsy (TRUS-Bx). The results showed that the use of povidone-iodine, in combination with ciprofloxacin, significantly reduced febrile complications, with a reduction from 15.38% to 2% in patients treated with both treatments. This study highlights the importance of combining effective disinfectants with antibiotic treatments to prevent infections in high-risk settings.

### 3.4. Comparative Analysis of Disinfectant Efficacy

The comparative analysis of disinfectants used to disinfect ultrasound probes highlighted significant differences in antimicrobial effectiveness and practical applicability, depending on the type of disinfectant and the pathogen in question. Various experimental studies and guidelines were considered to determine which disinfectant is most effective in preventing pathogen transmission between patients while preserving the functionality of ultrasound probes. The results from these studies show variability in effectiveness, with some disinfectants demonstrating clear superiority, especially against viral pathogens, while others are more effective against bacteria.

The study by Rutala et al. [57] demonstrated that the Trophon EPR system, which uses 35% H_2_O_2_, is highly effective against a range of pathogens, including resistant bacteria such as VRE (vancomycin-resistant enterococci) and *Mycobacterium terrae*, with a logarithmic reduction of over 6-log_10_ for VRE and over 5-log_10_ for *Mycobacterium terrae*. Additionally, the study by Meyers et al. [55] confirmed the effectiveness of H_2_O_2_ in reducing the infectivity of HPV16 by over 4-log_10_ and HPV18 by over 5-log_10_, whereas OPA a showed much lower reduction (<0.6-log_10_), indicating the superior effectiveness of H_2_O_2_, particularly against resistant viral pathogens like HPV. This result is crucial for probes used in transvaginal ultrasounds, where viral disinfection is essential [43,45].

Another study compared 4% chlorhexidine gluconate with other disinfectants for removing bacterial contaminants from ultrasound probes. Chlorhexidine gluconate was found to eliminate coagulase-negative *Staphylococcus* and *Bacillus* spp. in 84.62% of cases, showing good efficacy against bacteria. However, its action against viral pathogens, such as HPV, remains uncertain, and no sufficient data have been reported regarding its ability to inhibit high-risk viruses like HPV [45].

Glutaraldehyde, used in concentrations ranging from 2.4% to 3.2%, has also been examined for ultrasound probe disinfection. While it is effective against bacterial contamination, the study by M’Zali et al. [54] found that 13% of samples treated with glutaraldehyde were still contaminated with HPV DNA, suggesting that this disinfectant may not be fully effective against viruses. Furthermore, glutaraldehyde can damage ultrasound probes, making it less suitable for long-term use in certain clinical settings [48,49].

Dimethyl ammonium chloride (4%) resulted in a 73.08% reduction in bacterial contamination, according to the study by Whitehead et al. [56]. While it is useful for reducing bacterial contamination, there is insufficient data regarding its ability to eliminate viral pathogens like HPV, limiting its applicability in situations where resistant viruses need to be eliminated [45].

Sodium hypochlorite has been recommended in some guidelines for preventing viral infections, such as COVID-19. However, other guidelines advise against using this disinfectant on ultrasound probes, particularly on those for intracavitary applications, due to the risk of damaging the probe material. This compromises its practical utility in daily situations, despite its powerful antimicrobial action [47,48].

Another study, conducted by Sherman et al. [50], evaluated the effectiveness of 70% isopropyl alcohol for cleaning ultrasound probes. The results showed a complete (100%) reduction in bacterial contamination, with no evidence of viral contamination. This suggests that isopropyl alcohol is effective in reducing bacterial contamination risks, but its action against viruses was not explicitly tested in this study.

In the study by Meyers et al. [52], chlorine dioxide showed extremely potent antiviral activity, with a reduction in viral infectivity greater than 99.99% for HPV16 and HPV18. The results are comparable to the effectiveness of sodium hypochlorite, and chlorine dioxide proved to be an ideal choice for the disinfection of ultrasound probes, especially in settings with a high incidence of viral infections.

According to the study by Casalegno et al. [51], quaternary ammonium compounds in wipes are effective in reducing bacterial contamination, but 3% of the samples treated with these wipes were still contaminated with high-risk HPV. These data suggest that quaternary ammonium compounds, while effective against bacteria, may not provide complete protection against viral pathogens.

Below is a summary of the effectiveness and applicability of the different disinfectants used to disinfect ultrasound probes (Table 2). Each disinfectant was tested against specific pathogens, and the results are described in terms of effectiveness in reducing bacterial and viral contamination, as well as the limitations and recommendations regarding its use in clinical settings.

**Figure 2:**
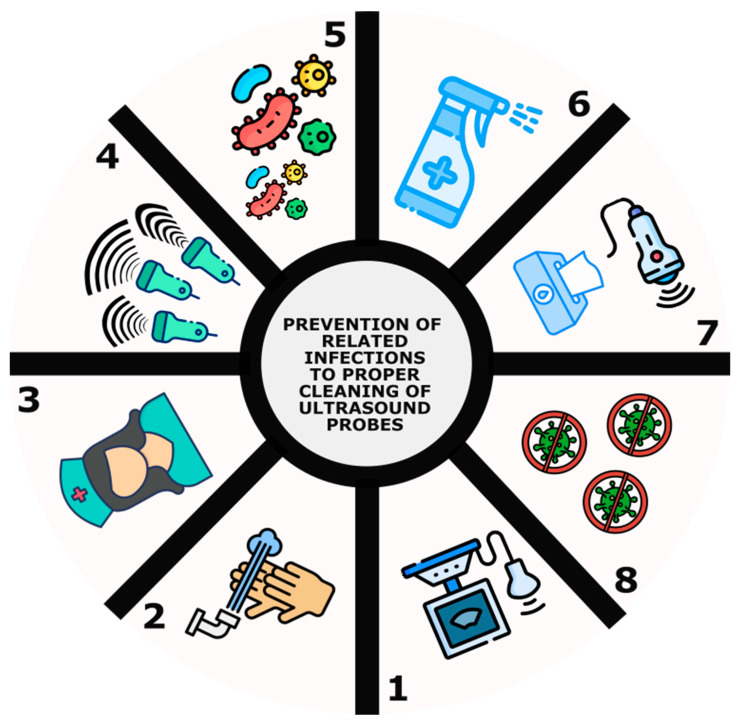
Process of ultrasound probe-associated infection Prevention. **Legend**: The figure illustrates the steps involved in the proper disinfection of ultrasound probes to prevent infections, ensuring the elimination of pathogens and reducing the risk of cross-contamination between patients.

Guidelines also diverge on the use of sodium hypochlorite. While some recommendations endorse its use for disinfection in the context of COVID-19 prevention [48], others advise against its application on ultrasound probes, particularly those used for intracavitary procedures, due to the risk of probe damage [47]. This discrepancy may stem from differing priorities in disinfection protocols: during the COVID-19 pandemic, the focus was on preventing viral transmission via high-contact surfaces, whereas in gynecological and urological settings, protecting probes from bacterial contamination or resistant viruses like HPV remains critical (Figure 2).

## 4. Discussion

This study aimed to determine the most effective disinfectants to prevent pathogen transmission via ultrasound probes. It also sought to compare available disinfectants, evaluating their antimicrobial efficacy to determine which methods best ensure patient safety while maintaining probe functionality. In line with this goal, the findings obtained in this study are consistent with those of several previous studies, but the inclusion of additional research helps expand our understanding of the effectiveness of disinfectants for ultrasound probes. Various studies have contributed to confirming the importance of targeted approaches for probe disinfection, highlighting several disinfectants and techniques with characteristics of efficacy, safety, and compatibility with the equipment.

Studies such as that of Casalegno et al. [51] suggest that disinfectants based on quaternary ammonium compounds have good overall efficacy, but viral contamination, such as from high-risk HPV, may persist despite the use of such disinfectants. This result underscores the need for more targeted disinfection protocols, particularly in contexts where viral contamination is a concern. This aligns with the main objective of this study, which was to identify the most effective disinfectants for preventing pathogen transmission between patients while preserving probe functionality.

The results regarding H_2_O_2_ highlight its effectiveness, particularly when used in automated systems like Trophon^®^ EPR, which has demonstrated the high potency of H_2_O_2_ against a wide range of pathogens, including resistant viruses like HPV [32,43]. Previous studies have shown similar results, recognizing H_2_O_2_’s ability to significantly reduce viral load, making it essential for preventing cross-contamination, especially in high-risk environments such as those involving intracavitary procedures. Other studies have also emphasized the dual benefits of H_2_O_2_, which not only provides robust antimicrobial protection but also minimizes the risk of damage to ultrasound probes, making it a safe and long-term solution for infection control [50,58].

Regarding chlorhexidine gluconate, the results confirmed its strong bactericidal properties, making it a valid option for reducing bacterial contamination. However, its limited effectiveness against viral pathogens like HPV suggests that its use should be more targeted, particularly in cases where bacterial contamination is the primary concern [45]. This finding, along with that of Siyez et al. [53] which highlighted the role of povidone-iodine in reducing infectious complications in patients undergoing transrectal prostate biopsy, suggests that different disinfectants may be needed depending on the nature of the pathogen. The combined use of disinfectants may therefore be particularly useful for addressing both bacterial and viral contamination [59].

Although glutaraldehyde is a broad-spectrum disinfectant, it has shown some limitations, particularly concerning damage to ultrasound probes. While glutaraldehyde has been successfully used in high-level disinfection protocols, the results of this study suggest that H_2_O_2_ is increasingly preferred in international guidelines due to its better balance between antimicrobial efficacy and safety for equipment [47,48,60,61].

In terms of technological innovation, the adoption of automated systems such as Trophon^®^ EPR and technologies like UVC radiation is showing considerable potential in ultrasound probe disinfection. Meyers et al. [52,55] demonstrated the effectiveness of UVC radiation in reducing the infectivity of HPV16 and HPV18, suggesting that this technology could be a promising solution, particularly in clinical settings. Furthermore, Rutala et al. [57] confirmed that the Trophon EPR system with 35% H_2_O_2_ is extremely effective against resistant pathogens, demonstrating greater than 6-log_10_ efficacy against VRE and over 5-log_10_ efficacy against *Mycobacterium terrae*. These results emphasize the importance of standardization and automation in probe disinfection, reducing the risk of human error and improving safety for both patients and healthcare workers [43,44,62,63,64].

However, despite the proven efficacy of these disinfection technologies, it is important to consider not only the chemical agents but also the mechanical aspects of cleaning ultrasound probes. Biofilm formation on probes is a significant concern, as microorganisms can form protective layers on the surface, making them more difficult to eliminate with disinfectants alone [65]. Therefore, mechanical cleaning methods, such as scrubbing or using specialized cleaning devices, are essential for the complete removal of biofilms and to enhance the overall effectiveness of chemical disinfection. For instance, a study demonstrated that the combination of air-polishing and cold atmospheric pressure plasma (CAP) was the most effective approach to biofilm removal from titanium surfaces, even several days after treatment [66]. Consequently, a combined strategy involving both chemical and mechanical methods is crucial to ensure the highest level of patient safety and to maintain the functionality of ultrasound probes.

Sherman et al. [50] confirmed that 70% alcohol is a valid option for ultrasound probe disinfection, effectively reducing bacterial contamination. However, since it has not been tested for viral disinfection, alcohol’s use may be limited in scenarios where viral contamination is predominant. These results are consistent with those reported by M’Zali et al. [54], who suggested that for complete protection against viruses like HPV, disinfectants with greater virucidal efficacy are needed.

Finally, the study by Whitehead et al. [56] highlighted the importance of an educational program for healthcare personnel, as targeted education significantly reduced bacterial contamination on Doppler probes. This emphasizes the critical role of training and education for healthcare workers in improving disinfection practices.

Despite the availability of effective disinfectants, consistent adherence to protocols remains challenging, as evidenced by incomplete compliance in several studies [46]. Ensuring that healthcare workers are adequately trained in the correct use of disinfectants, as well as in maintaining rigorous hand hygiene and the proper cleaning of high-contact surfaces, is crucial. Effective training programs, supported by routine assessments and feedback, can help embed best practices into daily clinical workflows, ultimately enhancing patient safety and reducing the risk of healthcare-associated infections [64,67]. Finally, it is important to consider that while disinfectants are crucial for infection control, their use can have adverse effects on both healthcare professionals and patients, as well as on the environment. For example, a study found that nurses exposed to cleaning and disinfecting agents, such as bleach and glutaraldehyde, had an increased risk of developing asthma and bronchial hyperresponsiveness (BHR)-related symptoms [68]. In addition to these health risks, certain disinfectants also pose environmental hazards. In terms of sustainability, peracetic acid stands out as the most environmentally friendly option due to its rapid biodegradation and minimal environmental footprint, making it a preferred choice when environmental concerns are prioritized [69,70].

### 4.1. Future Directions

This study underscores the critical need for further research to deepen our understanding of the comparative efficacy of disinfectants against a broader spectrum of pathogens, encompassing both bacterial and viral contaminants. As healthcare environments continue to adapt to the challenges posed by emerging infectious diseases, ongoing investigations are imperative to identify disinfectants that provide the most robust and comprehensive protection across diverse clinical settings. Future research should prioritize addressing barriers to protocol adherence by exploring innovative strategies, such as the integration of real-time monitoring systems and automated disinfection technologies, to enhance compliance and optimize infection control practices. Additionally, rigorous evaluations of the cost-effectiveness and environmental sustainability of disinfection methods are essential. Such studies will ensure that selected solutions are not only efficacious but also economically viable and ecologically responsible, supporting their implementation in long-term infection control strategies.

### 4.2. Study Limitations

This review has several limitations that should be acknowledged. This study is a “narrative” review of the literature and, as such, does not rely on a standardized methodology and may not be fully representative of all studies in the field, implying the possible exclusion of studies relevant to the topic with respect to the inclusion criteria. The data analyzed were derived from existing studies, which may vary in methodology, potentially affecting the comparability of results across different research groups. Additionally, this review predominantly focused on transvaginal ultrasound probes, limiting the generalizability of the findings to other types of ultrasound probes or clinical contexts. Future research should aim to encompass a broader range of probe types and clinical settings to offer a more comprehensive assessment of disinfectant efficacy. Furthermore, while this study concentrated on the antimicrobial effectiveness of the disinfectants, other critical factors such as cost-effectiveness, environmental impact, and ease of use were not evaluated and should be considered in future studies.

## 5. Conclusions

This study provided a comprehensive overview of the effectiveness of various disinfectants in disinfecting ultrasound probes, with a particular emphasis on preventing the transmission of pathogens between patients. Among the key findings, H_2_O_2_ stood out as the most effective, particularly when used in automated systems, while chlorhexidine gluconate demonstrated strong antibacterial capacity but showed limitations against viral pathogens. Although glutaraldehyde remains effective, it poses a higher risk of probe damage, underscoring the need to balance efficacy with equipment preservation. The practical implications of this study suggest that integrating automated disinfection systems, along with enhanced staff training, could improve disinfection effectiveness and compliance with protocols, thereby reducing the risk of healthcare-associated infections. Looking forward, future research should focus on the use of disinfectants in diverse clinical settings and explore innovative technological solutions that ensure both effective disinfection and greater economic and environmental sustainability.

## Figures and Tables

**Table 1:** General Characteristics of the Studies Included.

Author, Year, Country	Design	Sample	Type of Probe	Objective	Disinfectant	Method	Results
Westerway SC et al. [32], 2016, Australia	Clinical Trial	171 swabs	TA and TV	Prevalence of bacterial contamination on ultrasound probes	Alcohol-based wipes, glutaraldehyde 2.4%	LLD: alcohol-based wipe; HLD: glutaraldehyde 2.4%	LLD: 3% contamination on TA, 4% on TV; HLD: No contamination on either probe.
Vickery K et al. [44] 2013, Australia	Clinical Trial	Tested against 21 species	Intracavitary probe	Efficacy of H_2_O_2_-based disinfectant in Trophon device	Automated H_2_O_2_, glutaraldehyde	Efficacy test with M. terrae as test organism	Device met HLD and sporicidal criteria for all tested standards.
Ienghong K et al. [45] 2020, Thailand	RCT	104 ultrasound probes	Intracavitary probe (TV, endocavitary)	Comparison of cleaning methods for bacterial contamination	Dry wipe, liquid soap, 4% chlorhexidine, dimethyl ammonium chloride	Random assignment of methods	Chlorhexidine: 84.62% reduction in bacterial contamination; Ammonium chloride: 73.08%; Liquid soap: 30.77%; Dry wipe: 7.69%.
Lucet JC et al. [46] 2019, France	Observational Study	676 TVS procedures	TVS	Frequency of HPV detection on TV ultrasound probes	HLD: glutaraldehyde, H_2_O_2_, peracetic acid; LLD: quaternary ammonium and detergent	Probe disinfection during TVS procedures	HPV detected on 0.3% of ultrasound keyboards; No trace on probes or gel.
Ryndock E et al. [43] 2016, USA	Clinical Trial	N.A	Common ultrasound probe	Comparison of OPA and H_2_O_2_ devices against HPV16 and HPV18	OPA, H_2_O_2_	Efficacy test with 0.55% OPA and 35% and 31.5% H_2_O_2_	H_2_O_2_: >5-log_10_ reductions; OPA: <0.6-log_10_ reductions (ineffective).
ECRI [49] 2018, USA	Guideline	N.A	Probe classification	Recommendations for disinfection of ultrasound probes	Wipes/sprays with quaternary ammonium, H_2_O_2_, bleach	FDA-approved products per manufacturer’s instructions	Emphasizes importance of pre-cleaning and rinsing for efficacy.
Basseal JM et al. [48] 2020, Australia	Guideline	N.A	All types of ultrasound	Preventing infections during COVID-19 pandemic	70% ethanol, 0.5% H_2_O_2_, 0.1% sodium hypochlorite, 0.05–0.2% benzalkonium chloride	Two-step process: cleaning with detergent; disinfecting virucidal products or sodium hypochlorite	Strict disinfection measures essential during COVID-19.
Abramowicz JS et al. [47] 2017, USA	Guideline	N.A	TVS	Guidelines for cleaning and disinfection of TVS and TR probes	2.4–3.2% glutaraldehyde, ClO_2_, H_2_O_2_, sodium hypochlorite	Glutaraldehyde replaced by ClO_2_ and H_2_O_2_; sodium hypochlorite not recommended	Trophon EPR: 7 min disinfection; Glutaraldehyde: 5 min; Cidex: 20 min.
Casalegno JS et al. [51] 2012, France	Cross-Sectional Study	217 before and 200 after ultrasound examination	TSV	Effectiveness of disinfection procedure for covered endocavity probes	Quaternary ammonium wipes	Wipes for cleaning and disinfection	LLD: 3% samples contaminated with HR-HPV before disinfection.
Sherman T et al. [50] 2015, USA	Observational Study	26 outpatients	Common ultrasound probe	Effect of skin disinfection and gel on contamination	70% isopropyl alcohol	Skin disinfection before injection	No contamination detected (0%); Skin disinfection reduced contamination significantly (OR = 21.0, *p* = 0.001).
Siyez E et al. [53] 2021, Türkiye	Retrospective Observational Study	112 patients undergoing TRUS-Bx	Transrectal ultrasound	Infectious complications in men undergoing TRUS-Bx with and without povidone-iodine	Povidone-iodine, ciprofloxacin	Transrectal injection with ciprofloxacin prophylaxis	Febrile complications: 15.38% with ciprofloxacin; 2% with both.
M’Zali F et al. [54] 2014, France	Prospective Study	300 samples	Endovaginal probes	Antimicrobial efficacy of LLD on microorganisms	Quaternary ammonium, chlorhexidine wipes	Cleaning with tissue, disinfection with wipes	HPV DNA: 13%; C. trachomatis: 20%; Mycoplasma: 8%.
Meyers C et al. [52] 2020, UK	In Vitro Experimental Study	N.A.	Nasendoscopes, endocavity probes	Efficacy of chlorine dioxide against HPV16 and HPV18	Chlorine dioxide solutions	Cleaning, disinfection, rinsing with 7% glycine	>99.99% reduction in HPV16/HPV18 infectivity.
Whitehead E et al. [56], 2006, UK	Interventional Study	10 samples	Doppler probes	Bacterial contamination and effect of staff education	Alcohol wipes	Doppler probes cleaned with alcohol wipes	Bacterial contamination before education: 26%; after education: 4.76% (χ^2^ *p* < 0.05).
Meyers C et al. [55] 2017, USA	In Vitro Experimental Study	N.A.	Endocavitary probes	Efficacy of high-intensity UVC radiation on HPV16 and HPV18	UVC radiation, OPA, sodium hypochlorite	UVC disinfection for 90 s, OPA and sodium hypochlorite as controls	UVC: >4-log_10_ reduction in HPV16, nearly 5-log_10_ in HPV18.
Rutala WA et al. [57] 2016, USA	Experimental Study	55 ultrasound probes	Surface and endocavitary	Effectiveness of Trophon EPR H_2_O_2_ mist system	Trophon EPR with H_2_O_2_	HLD process with misting system	>6-log_10_ reduction in VRE and carbapenem-resistant Klebsiella pneumoniae; >5-log_10_ reduction in *Mycobacterium terrae* and *C. difficile*.

**Legend:** LLD: lower-level disinfection; HLD: high-level disinfection; TA: trans-abdominal; TV: transvaginal; TVS: transvaginal ultrasound; HPV: Human Papillomavirus; OPA: ortho-phthalaldehyde; H_2_O_2_: hydrogen peroxide; ClO_2_: chlorine dioxide; N.A.: not applicable; RCT: Randomized controlled trial; TRUS-Bx: transrectal ultrasound-guided prostate biopsy; VRE: vancomycin-resistant Enterococcus; TR: transrectal; *M. terrae*: *Mycobacterium terrae*; HR-HPV: high-risk Human Papillomavirus; OR: odds ratio.

**Table 2:** Comparative effectiveness of disinfectants in contamination reduction and pathogen inactivation.

Disinfectant	Pathogens Tested	Contamination Reduction	Effectiveness Against Viruses	Effectiveness Against Bacteria	Time Point *
H_2_O_2_ (35%)	HPV16, HPV18, VRE, *Mycobacterium terrae*	>5-log_10_ (HPV16 and HPV18)	Excellent (4–5-log_10_ against HPV)	>6-log_10_ (VRE), >5-log_10_ (*Mycobacterium terrae*)	6–8 min
Chlorhexidine Gluconate (4%)	*Staphylococcus*, *Bacillus*	84.62% reduction	Uncertain (No HPV data)	84.62% reduction	NR
Glutaraldehyde (2.4–3.2%)	*Staphylococcus*, HPV (DNA)	Significant reduction (13% HPV)	Limited (13% HPV contamination)	Effective against bacteria	20 min
Dimethyl Ammonium Chloride (4%)	*Staphylococcus*, *Bacillus*	73.08% reduction	Not tested against viruses	73.08% reduction	NR
Sodium Hypochlorite	Various pathogens	Uncertain (probe damage risk)	Effective against viruses (probe damage)	Effective but damages probes	10 min
Isopropyl Alcohol (70%)	Bacteria (no viral testing)	100% reduction (bacteria)	Not tested against viruses	Excellent against bacteria	1–2 min
Chlorine Dioxide	HPV16, HPV18	>99.99% viral infectivity reduction	Excellent (>99.99% reduction)	Effective against bacteria	3–5 min
Quaternary Ammonium Compounds	*Staphylococcus*, HPV (high risk)	Bacterial reduction (3% HPV)	Limited (3% HPV contamination)	Effective against bacteria	10 min

**Legend**: * = Recommendations from the Centers for Disease Control and Prevention, Occupational Safety and Health Administration and Environmental Protection Agency (USA); NR = not reported; HPV = Human Papillomavirus; VRE = vancomycin-resistant Enterococcus; DNA = deoxyribonucleic acid; H_2_O_2_ = hydrogen peroxide.

## Data Availability

The data supporting this research are available upon request from the corresponding author for data protection reasons.

## Supplementary Materials

The following supporting information can be downloaded at https://www.mdpi.com/article/10.3390/microorganisms12122394/s1, Supplementary File S1: Search strategy.
