# Supplementary material for: Optimizing Ultrasound Probe Disinfection for Healthcare-Associated Infection Control: A Comparative Analysis of Disinfectant Efficacy
Source: Microorganisms. 2024 Nov 22;12(12):2394. doi: 10.3390/microorganisms12122394 (PMC11676816; doi:10.3390/microorganisms12122394)
Supplement: Supplementary file 1 [file microorganisms-12-02394-s001.zip › microorganisms-3315472-supplementary.pdf]

Table S1. Search Strategy

ID	Cochrane Library	Results
#1	disinfectant OR antiseptic OR "cleaning agent" OR "sterilizing agent" OR "disinfection solution" OR disinfection OR antiseptic OR "cleaning agents" OR "sterilizing agents" OR "disinfection solutions" OR sanitizer OR germicide OR sanitizers OR germicides	
#2	"ultrasound probes" OR "ultrasound transducers" OR "ultrasound devices" OR "ultrasound equipment" OR probe OR transducer OR "medical probe" OR "sensor probe" OR "diagnostic probe" OR "ultrasound probe" OR "ultrasound transducer" OR "ultrasound device" OR "ultrasound equipments" OR probes OR transducers OR "medical probes" OR "sensor probes" OR "diagnostic probes"	
#3	"pathogen transmission" OR "disease transmission" OR "infection spread" OR "cross-contamination" OR "microbial transmission" OR infection OR contamination OR "infectious disease" OR "infectious agent" OR "pathogenic infection" OR "pathogen transmissions" OR "disease transmissions" OR "infection spreads" OR "cross-contaminations" OR "microbial transmissions" OR infections OR contaminations OR "infectious diseases" OR "infectious agents" OR "pathogenic infections"	
**#4**	**#1 AND #2 AND #3**<br><br>**disinfectant OR antiseptic OR "cleaning agent" OR "sterilizing agent" OR "disinfection solution" OR disinfection OR antiseptic OR "cleaning agents" OR "sterilizing agents" OR "disinfection solutions" OR sanitizer OR germicide OR sanitizers OR germicides in Title Abstract Keyword AND "ultrasound probes" OR "ultrasound transducers" OR "ultrasound devices" OR "ultrasound equipment" OR probe OR transducer OR "medical probe" OR "sensor probe" OR "diagnostic probe" OR "ultrasound probe" OR "ultrasound transducer" OR "ultrasound device" OR "ultrasound equipments" OR probes OR transducers OR "medical probes" OR "sensor probes" OR "diagnostic probes" in Title Abstract Keyword AND "pathogen transmission" OR "disease transmission" OR "infection spread" OR "cross-contamination" OR "microbial transmission" OR infection OR contamination OR "infectious disease" OR "infectious agent" OR "pathogenic infection" OR "pathogen transmissions" OR "disease transmissions" OR "infection spreads" OR "cross-contaminations" OR "microbial transmissions" OR infections OR contaminations OR "infectious diseases" OR "infectious agents" OR "pathogenic infections" in Title Abstract Keyword - (Word variations have been searched)**	**Trial: 147**<br><br>**Reviews: 1**
	**Pubmed**	**Results**
#1	Disinfectants[Mesh] OR Disinfection[Mesh] OR disinfectant OR antiseptic OR "cleaning agent" OR "sterilizing agent" OR "disinfection solution" OR disinfection OR antiseptic OR "cleaning agents" OR "sterilizing agents" OR "disinfection solutions" OR sanitizer OR germicide OR sanitizers OR germicides	
#2	Ultrasonography[Mesh] OR "ultrasound probes" OR "ultrasound transducers" OR "ultrasound devices" OR "ultrasound equipment" OR probe OR transducer OR "medical probe" OR "sensor probe" OR "diagnostic probe" OR "ultrasound probe" OR "ultrasound transducer" OR "ultrasound device" OR "ultrasound equipments" OR probes OR transducers OR "medical probes" OR "sensor probes" OR "diagnostic probes"	
#3	Disease Transmission, Infectious[Mesh] OR "pathogen transmission" OR "disease transmission" OR "infection spread" OR "cross-contamination" OR "microbial transmission" OR infection OR contamination OR "infectious disease" OR "infectious	

	agent" OR "pathogenic infection" OR "pathogen transmissions" OR "disease transmissions" OR "infection spreads" OR "cross-contaminations" OR "microbial transmissions" OR infections OR contaminations OR "infectious diseases" OR "infectious agents" OR "pathogenic infections"	
**#4**	**#1 AND #2 AND #3**<br><br>**((Disinfectants[Mesh] OR Disinfection[Mesh] OR disinfectant OR antiseptic OR "cleaning agent" OR "sterilizing agent" OR "disinfection solution" OR disinfection OR antiseptic OR "cleaning agents" OR "sterilizing agents" OR "disinfection solutions" OR sanitizer OR germicide OR sanitizers OR germicides) AND (Ultrasonography[Mesh] OR "ultrasound probes" OR "ultrasound transducers" OR "ultrasound devices" OR "ultrasound equipment" OR probe OR transducer OR "medical probe" OR "sensor probe" OR "diagnostic probe" OR "ultrasound probe" OR "ultrasound transducer" OR "ultrasound device" OR "ultrasound equipments" OR probes OR transducers OR "medical probes" OR "sensor probes" OR "diagnostic probes")) AND (Disease Transmission, Infectious[Mesh] OR "pathogen transmission" OR "disease transmission" OR "infection spread" OR "cross-contamination" OR "microbial transmission" OR infection OR contamination OR "infectious disease" OR "infectious agent" OR "pathogenic infection" OR "pathogen transmissions" OR "disease transmissions" OR "infection spreads" OR "cross-contaminations" OR "microbial transmissions" OR infections OR contaminations OR "infectious diseases" OR "infectious agents" OR "pathogenic infections") Filters: from 2014 - 2024**	**787**
**ID**	**Cumulative Index Of Nursing And Allied Health Literature (CINAHL)**	**Results**
#1	disinfectant OR antiseptic OR "cleaning agent" OR "sterilizing agent" OR "disinfection solution" OR disinfection OR antiseptic OR "cleaning agents" OR "sterilizing agents" OR "disinfection solutions" OR sanitizer OR germicide OR sanitizers OR germicides	
#2	"ultrasound probes" OR "ultrasound transducers" OR "ultrasound devices" OR "ultrasound equipment" OR probe OR transducer OR "medical probe" OR "sensor probe" OR "diagnostic probe" OR "ultrasound probe" OR "ultrasound transducer" OR "ultrasound device" OR "ultrasound equipments" OR probes OR transducers OR "medical probes" OR "sensor probes" OR "diagnostic probes"	
#3	"pathogen transmission" OR "disease transmission" OR "infection spread" OR "cross-contamination" OR "microbial transmission" OR infection OR contamination OR "infectious disease" OR "infectious agent" OR "pathogenic infection" OR "pathogen transmissions" OR "disease transmissions" OR "infection spreads" OR "cross-contaminations" OR "microbial transmissions" OR infections OR contaminations OR "infectious diseases" OR "infectious agents" OR "pathogenic infections"	
**#4**	**#1 AND #2 AND #3**<br><br>**(disinfectant OR antiseptic OR "cleaning agent" OR "sterilizing agent" OR "disinfection solution" OR disinfection OR antiseptic OR "cleaning agents" OR "sterilizing agents" OR "disinfection solutions" OR sanitizer OR germicide OR sanitizers OR germicides) AND ("ultrasound probes" OR "ultrasound transducers" OR "ultrasound devices" OR "ultrasound equipment" OR probe OR transducer OR "medical probe" OR "sensor probe" OR "diagnostic probe" OR "ultrasound probe" OR "ultrasound transducer" OR "ultrasound device" OR "ultrasound equipments" OR probes OR transducers OR "medical probes" OR "sensor probes" OR "diagnostic probes") AND ("pathogen transmission" OR "disease transmission" OR "infection spread" OR "cross-contamination" OR "microbial transmission" OR infection OR**	**107**

	**contamination OR "infectious disease" OR "infectious agent" OR "pathogenic infection" OR "pathogen transmissions" OR "disease transmissions" OR "infection spreads" OR "cross-contaminations" OR "microbial transmissions" OR infections OR contaminations OR "infectious diseases" OR "infectious agents" OR "pathogenic infections")**	
**ID**	**EMBASE**	**160**
#1	disinfectant OR antiseptic OR "cleaning agent" OR "sterilizing agent" OR "disinfection solution" OR disinfection OR antiseptic OR "cleaning agents" OR "sterilizing agents" OR "disinfection solutions" OR sanitizer OR germicide OR sanitizers OR germicides	
#2	"ultrasound probes" OR "ultrasound transducers" OR "ultrasound devices" OR "ultrasound equipment" OR probe OR transducer OR "medical probe" OR "sensor probe" OR "diagnostic probe" OR "ultrasound probe" OR "ultrasound transducer" OR "ultrasound device" OR "ultrasound equipments" OR probes OR transducers OR "medical probes" OR "sensor probes" OR "diagnostic probes"	
#3	"pathogen transmission" OR "disease transmission" OR "infection spread" OR "cross-contamination" OR "microbial transmission" OR infection OR contamination OR "infectious disease" OR "infectious agent" OR "pathogenic infection" OR "pathogen transmissions" OR "disease transmissions" OR "infection spreads" OR "cross-contaminations" OR "microbial transmissions" OR infections OR contaminations OR "infectious diseases" OR "infectious agents" OR "pathogenic infections"	
**#4**	**#1 AND #2 AND #3**<br><br>**(disinfectant:ti,ab,kw OR 'cleaning agent':ti,ab,kw OR 'sterilizing agent':ti,ab,kw OR 'disinfection solution':ti,ab,kw OR disinfection:ti,ab,kw OR antiseptic:ti,ab,kw OR 'cleaning agents':ti,ab,kw OR 'sterilizing agents':ti,ab,kw OR 'disinfection solutions':ti,ab,kw OR sanitizer:ti,ab,kw OR germicide:ti,ab,kw OR sanitizers:ti,ab,kw OR germicides:ti,ab,kw) AND ('ultrasound probes':ti,ab,kw OR 'ultrasound transducers':ti,ab,kw OR 'ultrasound devices':ti,ab,kw OR 'ultrasound equipment':ti,ab,kw OR probe:ti,ab,kw OR transducer:ti,ab,kw OR 'medical probe':ti,ab,kw OR 'sensor probe':ti,ab,kw OR 'diagnostic probe':ti,ab,kw OR 'ultrasound probe':ti,ab,kw OR 'ultrasound transducer':ti,ab,kw OR 'ultrasound device':ti,ab,kw OR 'ultrasound equipments':ti,ab,kw OR probes:ti,ab,kw OR transducers:ti,ab,kw OR 'medical probes':ti,ab,kw OR 'sensor probes':ti,ab,kw OR 'diagnostic probes':ti,ab,kw) AND ('pathogen transmission':ti,ab,kw OR 'disease transmission':ti,ab,kw OR 'infection spread':ti,ab,kw OR 'cross-contamination':ti,ab,kw OR 'microbial transmission':ti,ab,kw OR infection:ti,ab,kw OR contamination:ti,ab,kw OR 'infectious disease':ti,ab,kw OR 'infectious agent':ti,ab,kw OR 'pathogenic infection':ti,ab,kw OR 'pathogen transmissions':ti,ab,kw OR 'disease transmissions':ti,ab,kw OR 'infection spreads':ti,ab,kw OR 'cross-contaminations':ti,ab,kw OR 'microbial transmissions':ti,ab,kw OR infections:ti,ab,kw OR contaminations:ti,ab,kw OR 'infectious diseases':ti,ab,kw OR 'infectious agents':ti,ab,kw OR 'pathogenic infections':ti,ab,kw) AND [2014-2024]/py**	
	**TOTAL ARTICLES FOUND**	**1.202**
